# The Effect of Mild Renal Dysfunction on the Assessment of Plasma Amino Acid Concentration and Insulin Resistance in Patients with Type 2 Diabetes Mellitus

**DOI:** 10.1155/2022/2048300

**Published:** 2022-06-13

**Authors:** Hideki Ikeda

**Affiliations:** Department of Internal Medicine, Sanyudo Hospital, Chuo 6 Chome-1-219, Yonezawa, Yamagata 992-0045, Japan

## Abstract

**Background:**

An increase in the levels of branched-chain amino acids (BCAAs) and certain aromatic amino acids, such as alanine, in plasma is correlated with insulin resistance (IR) in type 2 diabetes mellitus (T2DM). T2DM is a leading risk factor for chronic kidney disease. Meanwhile, renal dysfunction causes changes in plasma amino acid levels. To date, no study has examined how mild renal dysfunction and IR interact with plasma amino acid levels. This study examines the effects of IR and renal dysfunction on plasma amino acid concentrations in T2DM.

**Methods:**

Data were collected from healthy male participants (controls) and male patients with T2DM between May 2018 and February 2022. Blood samples were collected after overnight fasting. IR and renal function were evaluated using the homeostasis model assessment of IR (HOMA-IR) and serum cystatin C (CysC), respectively.

**Results:**

A total of 49 and 93 participants were included in the control and T2DM groups, respectively. In the T2DM group, eight amino acids (alanine, glutamic acid, glutamine, glycine, isoleucine, leucine, tyrosine, and valine) and total BCAA showed a significant correlation with HOMA-IR (*p* < 0.01), whereas six amino acids (*γ*-aminobutyric acid, citrulline, cysteine, glycine, methionine, and valine) and total BCAA showed a significant correlation with 1/CysC (*p* < 0.02). However, only alanine, glutamic acid, and each BCAA showed significant differences between the control group and the IR T2DM subgroup. Increases in the BCAA levels with T2DM were canceled by renal dysfunction (CysC ≥ 0.93) in patients with intermediate IR.

**Conclusion:**

To use plasma BCAA concentration as a marker of IR, renal function must be considered, even in mild renal dysfunction. Increased alanine and glutamic acid levels indicate IR, regardless of mild renal dysfunction.

## 1. Introduction

Diabetes mellitus (DM), an abnormality in glucose metabolism, is also known to affect amino acid metabolism, particularly by altering the plasma concentration of glutamic acids, branched-chain amino acids (BCAAs), and aromatic amino acids (AAAs) [1–6]. Among these three types of amino acids, glutamic acid is correlated with metabolic risks [7], and the metabolism of glutamic acid, *γ*-aminobutyric acid (GABA), and glutamine plays a role in the regulation of insulin and glucagon [8]. Furthermore, an increase in the AAA tyrosine has been reported to be correlated with insulin resistance (IR) [9–11]. In addition, in recent years, there has been a rapid increase in the number of research reports on the correlation between BCAAs and IR [4, 11–15]. DM is a leading cause of end-stage renal dysfunction worldwide [16], including in Japan, where it is also a leading risk factor for chronic kidney disease (CKD) [17]. Furthermore, the kidneys play an essential role in amino acid metabolism [18–22], and CKD causes essential amino acids, including BCAAs, to decrease and nonessential amino acids to increase [21, 23]. Even mild renal dysfunction causes plasma amino acid levels, including BCAA levels, to begin to change [24, 25]. Furthermore, as the kidneys are also responsible for metabolizing phenylalanine into tyrosine [26], CKD may also affect IR through the changes it causes during this process [26].

Unlike other amino acids, BCAAs undergo primary metabolism in muscle tissue. They are metabolized to branched-chain *α*-keto acids (BCKAs) and released into the bloodstream before being metabolized by the liver and other tissues [20, 27]. At this point, the kidneys turn the BCKAs back into BCAAs, which play an important role in the homeostasis of blood BCAA concentrations [20, 22]. In type 2 diabetes mellitus (T2DM) with IR, there is decreased enzyme activity in the metabolic pathways of isoleucine and valine [13] and decreased systemic leucine metabolism [14], which is believed to increase plasma BCAA concentrations. This increase is in contrast with the decrease in plasma BCAA concentrations observed in CKD due to the impaired regeneration of BCAAs. Consequently, there is a possibility that these two changes in plasma BCAA concentrations will cancel each other out when IR and mild renal dysfunction cooccur. However, no studies have examined how mild renal dysfunction and IR interact with plasma amino acid levels. To that end, this study is aimed at examining the effects of IR and renal dysfunction on plasma amino acid concentrations in T2DM, with the homeostasis model assessment of IR (HOMA-IR) [28–30] as a marker of IR and serum cystatin C (CysC) [31–34] as an indicator of renal function assessment.

## 2. Patients and Methods

For the T2DM group, male patients with T2DM who attended the Sanyudo Hospital regularly for outpatient care between January and August 2021 were selected. Criteria for inclusion were glycated hemoglobin (HbA1c) ≥ 6.2% and taking blood glucose medication. However, patients undergoing treatment by insulin injection, patients taking systemic steroid hormones, and patients with thyroid dysfunction, viral hepatitis, or malignant disorders were excluded. Healthy male participants (control group) included those who underwent a medical examination between May 2018 and February 2022 at the Sanyudo Hospital. However, participants, who were on medication for hyperlipidemia, had DM (HbA1c ≥ 6.2% or were on medication for DM), had kidney dysfunction (estimated glomerular filtration rate (eGFR) < 60 mL/min/1.73 m^2^), or were determined to have a malignant disorder were excluded from the analysis.

Fasting began at 9 : 00 PM the previous night, and venous blood samples were collected between 8 : 00 and 9 : 30 AM from both groups. Samples for measuring blood urea nitrogen (BUN) and serum creatinine (Cr) were collected in quick-clotting test tubes (containing thrombin and thrombin-like enzymes), whereupon the serum was separated. BUN was measured using the urease-GLDH/ICDH/UV method (ammonia elimination). Cr was measured using an enzymatic method (SOX-POD type). eGFR was calculated using the following formula: 194 × Cr^−1.094^ × age^−0.287^ [35]. Samples for amino acid analysis were placed in test tubes containing EDTA-2Na immediately after collection and were stored in ice. Thereafter, they were centrifuged, and the plasma was cryopreserved at -40°C. Plasma amino acid was measured using a liquid chromatography-mass spectrometer (SRL Inc., Tokyo, Japan). Additionally, CysC, fasting serum insulin concentration (fasting IRI), and fasting blood glucose (FBS) concentration were measured in the patients of the T2DM group. Samples for measuring FBS were collected in test tubes containing sodium fluoride, and FBS was measured using the glucose oxidase immobilized electrode method. For measuring CysC and fasting IRI, samples were collected in test tubes containing a clotting accelerator (silica) and preserved at 4°C after serum separation. CysC and fasting IRI were measured using the colloidal gold agglutination method and the chemiluminescent enzyme immunoassay method, respectively. HOMA-IR was determined according to the following formula: HOMA − IR = FBS (mg/dL) × fasting IRI (*μ*U/mL)/405.

This study was conducted following approval at the 57th, 59th, 63rd, 65th, and 70th Sanyudo Hospital Ethics Committee meetings. In addition, informed consent was obtained from participants in writing, in accordance with the Declaration of Helsinki. These processes were conducted in accordance with the Ministry of Health, Labour, and Welfare's Ethical Guidelines for Medical and Health Research Involving Human Subjects [36].

### 2.1. Statistical Analysis

A Mann–Whitney *U*-test was performed to compare the control and T2DM groups. A nonparametric multiple comparison test (Steel–Dwass method) was used to compare the control group with the T2DM subgroups classified by HOMA-IR and CysC. The correlations between eGFR and 1/CysC and between HbA1c and HOMA-IR were found using the least squares method. The correlations of each plasma amino acid level with age and eGFR in the control group and with age, HOMA-IR, and 1/CysC in the T2DM group were analyzed using multiple regression analysis. In addition, the distribution of patients with hypertension or diabetic retinopathy was analyzed using chi-square test.

## 3. Results

The control and T2DM groups included 49 and 93 participants, respectively, with mean ages of 65.9 ± 10.2 and 67.2 ± 10.2 years, respectively. No significant difference in age was noted between the two groups (*p* = 0.475). The correlations between each amino acid level and age and eGFR in the control group are shown in Table 1. Taurine, aspartic acid, and cystine levels significantly correlated with age (*p* < 0.01). However, none of the amino acid levels in the control group were found to correlate with eGFR.

Table 2 shows the medications dispensed to patients with T2DM. Biguanide or/and dipeptidyl peptidase 4 inhibitor were mainly dispensed, and others were combined with these two. Only 11 (11.8%) patients were treated with single medication.  Table 3 shows the distribution of HbA1c and body mass index (BMI) in the T2DM group. HbA1c and HOMA-IR had a weak correlation (*r* = 0.3127, *p* = 0.0023). BMIs were mainly distributed in the normal range (51%) and obese 1 (29%) according to Japanese classification [37]. Sixty-four (68.8%) patients had hypertension (HT), and 19 (20.4%) patients had diabetic retinopathy (DR) as comorbidities.

**Figure 1:**
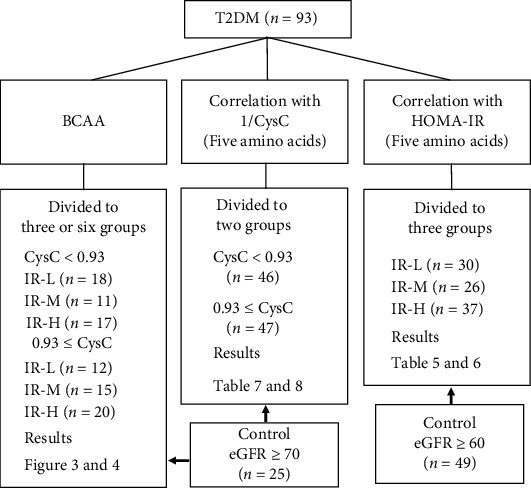
Flowchart of the classification and number of participants. T2DM: type 2 diabetes mellitus; BCAAs: branched-chain amino acids; CysC: cystatin C; HOMA-IR: homeostasis model assessment of insulin resistance; IR-L, T2DM with HOMA − IR ≤ 1.60; IR-M, T2DM with 1.6 < HOMA − IR < 2.5; IR-H, T2DM with HOMA − IR ≥ 2.5; eGFR: estimated glomerular filtration rate.

Table 4 shows the results of the multiple correlation analysis between each amino acid level and age, HOMA-IR, and 1/CysC in the T2DM group. Leucine and valine levels significantly correlated with age; therefore, they were adjusted to a mean age of 67.2 years in subsequent analyses. In the T2DM group, eight amino acids (alanine, glutamic acid, glutamine, glycine, isoleucine, leucine, tyrosine, and valine) and total BCAA showed a significant correlation with HOMA-IR (*p* < 0.01), whereas six amino acids (GABA, citrulline, cysteine, glycine, ornithine, and valine) and total BCAA showed a significant correlation with 1/CysC (*p* < 0.05). Therefore, amino acids, which had significant correlation with HOMA-IR or 1/CysC, were selected for comparison with the control group. Namely, amino acids were classified to BCAAs, amino acids that had correlation with 1/CysC except BCAA, amino acids that had correlation with HOMA-IR except BCAA (Figure 1).

**Figure 2:**
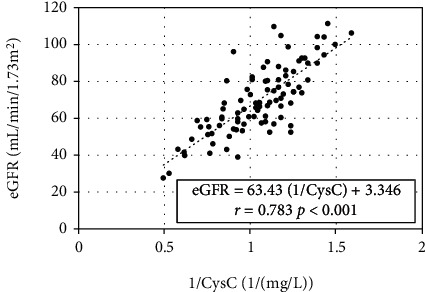
Correlation between 1/CysC and eGFR in T2DM. The correlation was calculated using the least-squares method. CysC; cystatin C; eGFR: estimated glomerular filtration rate; T2DM: type 2 diabetes mellitus.

A good linear correlation was found between eGFR and 1/CysC (Figure 2). On the regression line, the CysC values corresponding to eGFRs of 60 and 90 mL/min/1.73 m^2^ were 1.120 and 0.732, respectively. In the T2DM group, 15 patients (23.4%) had moderate or greater renal dysfunction (CysC > 1.120, corresponding to eGFR < 60 mL/min/1.73 m^2^ [38]), 70 patients (68.1%) had mild renal dysfunction (0.732 < CysC < 1.120, corresponding to 60 < eGFR < 90 mL/min/1.73 m^2^ [38]), and 8 patients (8.5%) had normal renal function (CysC < 0.732, corresponding to eGFR > 90 mL/min/1.73 m^2^ [38]). As the median CysC value in the T2DM group was 0.925, the value of 0.93 was used to divide the group into a good renal function subgroup (*n* = 46) and a decreased renal function subgroup (*n* = 47) for this study. On the regression line, the eGFR value corresponding to a CysC value of 0.93 was 71.5 mL/min/1.73 m^2^. Therefore, only those participants in the control group with eGFR ≥ 70 mL/min/1.73 m^2^ (*n* = 25) were included in the comparison with the CysC subgroups and in the comparison of BCAA levels in T2DM. For Japanese people, a HOMA − IR ≤ 1.6 indicates the absence of IR, whereas a HOMA − IR ≥ 2.5 indicates the presence of IR [29, 30]. Therefore, the T2DM group was divided into three subgroups according to the HOMA-IR values: IR-L, HOMA − IR ≤ 1.60; IR-M, 1.60 < HOMA − IR < 2.50; IR-H, HOMA − IR ≥ 2.50. Figure 1 shows the flowchart for this selection and the number of participants per group as well as the corresponding tables and figures. DR was present in 6, 7, and 6 participants in the IR-L, IR-M, and IR-H subgroups, respectively. Meanwhile, HT was present in 17, 21, and 26 participants in the IR-L, IR-M, and IR-H, respectively. There were no statistical significances among each subgroup (*p* = 0.181 for DR and *p* = 0.147 for HT). The number and frequency of patients with DR was significantly higher (*p* = 0.013) in the subgroup with high CysC than in the subgroup with low CysC (15 and 31.9% vs. 4 and 8.7%). The number and frequency of patients with HT was higher (*p* = 0.037) in the subgroup with high CysC than in the subgroup with low CysC (37 and 78.7% vs. 27 and 58.7%).

**Figure 3:**
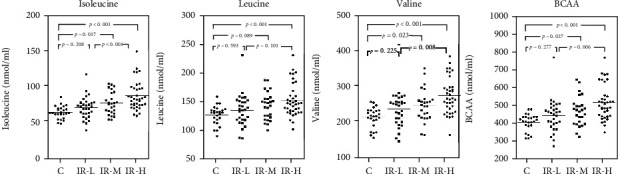
Intergroup comparisons for BCAAs in the control and T2DM groups. Statistical analysis was performed using the Steel–Dwass method. The horizontal solid lines indicate the mean values for each group. IR-L, T2DM with HOMA − IR ≤ 1.60; IR-M, T2DM with 1.6 < HOMA − IR < 2.5; IR-H, T2DM with HOMA − IR ≥ 2.5. BCAAs: branched-chain amino acids; T2DM: type 2 diabetes mellitus; HOMA-IR: homeostasis model assessment of insulin resistance.

**Figure 4:**
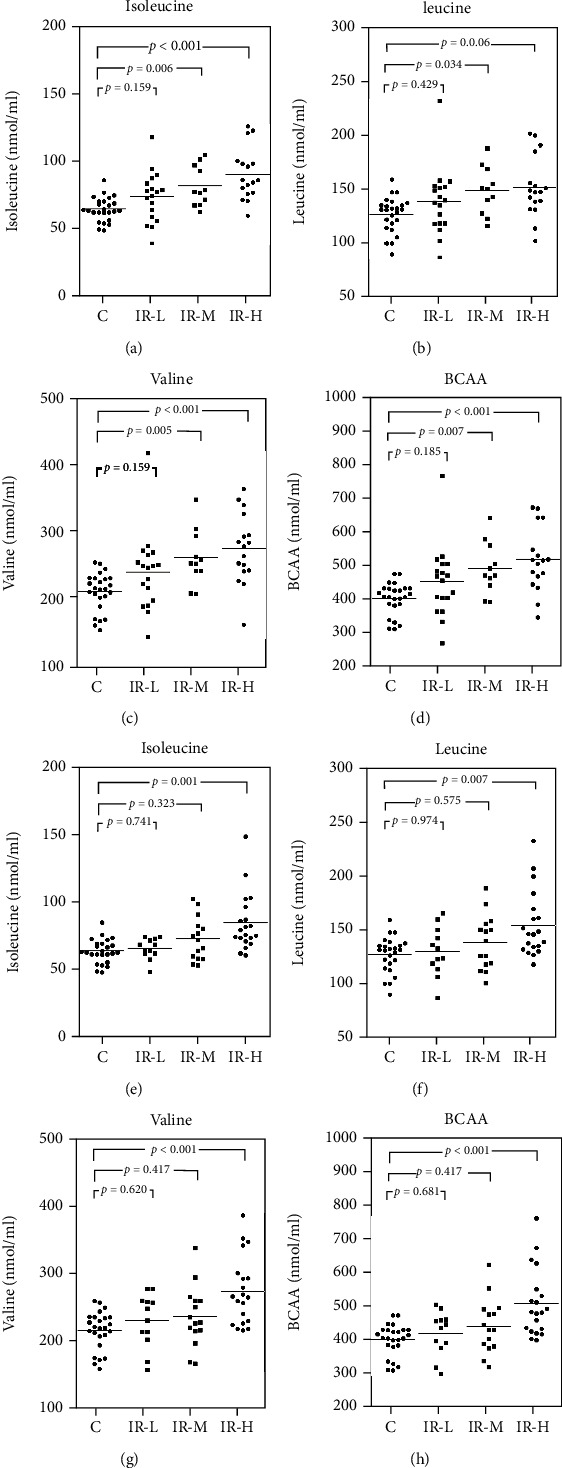
Intergroup comparisons for BCAAs in the control and T2DM groups. Statistical analysis was performed using the Steel–Dwass method. The horizontal solid lines indicate the mean values for each group. (a–d) CysC < 0.93; (e–h) Cys ≥ 0.93. IR-L, T2DM with HOMA − IR ≤ 1.60; IR-M, T2DM with 1.6 < HOMA − IR < 2.5; IR-H, T2DM with HOMA − IR ≥ 2.5. BCAAs: branched-chain amino acids; CysC: cystatin C; T2DM: type 2 diabetes mellitus; HOMA-IR: homeostasis model assessment of insulin resistance.

Figure 3 shows the comparison of each BCAA level in the control group (eGFR ≥ 70 mL/min/1.73 m^2^) with those in the T2DM HOMA-IR subgroups. Each BCAA level was significantly higher (*p* < 0.01) in the IR-H subgroup than in the control group and IR-L subgroup, except for leucine when comparing between the IR-L and IR-H subgroups. The IR-M subgroup showed significantly higher BCAA levels than the control group, except for leucine. Furthermore, there was no difference in the BCAA levels between the IR-L subgroup and the control group. Finally, each BCAA level was compared in the two T2DM CysC subgroups (cut-off value, 0.93) (Figure 4). In the subgroup with CysC < 0.93 (Figures 4(a)–4(d)), the differences in BCAAs between the control group and the IR-M or IR-H subgroup were similar to those presented in Figure 3. Furthermore, there was a significant difference in leucine between the control group and the IR-M subgroup (Figure 4(b)). However, the differences in BCAAs between the control group and the IR-M subgroup disappeared in the subgroup with CysC ≥ 0.93 (Figures 4(e)–4(h)).

Tables 5–8 show a comparison between the control group and the HOMA-IR or CysC subgroups with regard to the non-BCAA amino acids that were correlated with HOMA-IR or CysC, respectively. A significant difference in alanine and glutamic acid levels was found between the control and T2DM groups when the latter was divided based on HOMA-IR (Tables 5 and 6). Furthermore, these amino acids were also found to increase significantly along with an increase in HOMA-IR (Table 4). Meanwhile, no significant difference was found between the control and T2DM groups when the latter was divided based on CysC values (Tables 7 and 8). A significant difference was found in GABA between the control group and the low-CysC T2DM subgroup (*p* = 0.0372) and in cystine between the high- (CysC ≥ 0.93) and the low-CysC T2DM subgroups (CysC < 0.93) (*p* = 0.0249).

## 4. Discussion

In this study, 83.9% of patients in the T2DM group had mild renal dysfunction or normal function (CysC < 1.120, corresponding to eGFR > 60 mL/min/1.73 m^2^). Mild renal dysfunction is believed be in a “creatinine-blind area,” in which Cr is considered inappropriate as an indicator of renal function [39, 40]. Instead of Cr, measuring CysC levels has been proposed as a method for assessing renal dysfunction [32]. CysC, produced *in vivo* like Cr, is a serum protein that exists abundantly in body fluid and is produced at the same rate regardless of age [31]. Furthermore, CysC does not bind to proteins in the blood and is filtered by renal glomeruli. Thus, it is broken down into amino acids when reabsorbed by the proximal convoluted tubule and does not return to the bloodstream [32]. It is also useful as a diagnostic marker for mild renal dysfunction [34], as the serum CysC concentration depends on GFR [33]. Serum CysC concentration has also been reported to be an excellent marker of renal function in diabetic nephropathy (DN) [40, 41], making it appropriate for the assessment of renal function in groups with a high occurrence of mild renal dysfunction, as was the case in this study.

In this study, increased BCAA concentrations were observed in T2DM, and this increase was weakened when renal dysfunction was present (Figure 4). In other words, even mild renal dysfunction was shown to impede the ability to assess IR using BCAA concentrations. Therefore, renal function must be considered using plasma BCAA concentration as a marker of IR, even in mild renal dysfunction. One report in the literature states that high leucine levels in DM lower the risk of DN [42]. However, this may reflect a decrease in BCAAs due to preexisting mild renal dysfunction in the creatinine-blind area. In recent years, there has been growing awareness of the involvement of BCAAs in the regulation of blood glucose, feeding center, and immune system [43]. How the canceling out of the increase in BCAAs correlates with IR and how the decrease in BCAAs due to CKD is involved in the feeding center and immune system should be further clarified.

Besides BCAAs, high levels of AAAs, alanine, glutamic acid, ornithine, and lysine have been reported to be markers of future T2D risk in healthy Japanese individuals [41]. In contrast, high levels of glutamine are considered to lower future T2D risk [41]. The AAAs phenylalanine and tyrosine have also correlated with DR and DN [44]. The results of this study do not contradict these findings, as alanine, glutamic acid, glycine, and tyrosine exhibited a positive correlation with HOMA-IR in T2DM (Table 4) and glutamine exhibited a negative correlation (Table 4). Particularly in alanine and glutamic acid, there was a significant difference between the control and IR-M or IR-H subgroups (Tables 5 and 6). Furthermore, they did not correlate with 1/CysC (Table 4). This indicates that alanine and glutamic acid could be used as markers for IR regardless of mild renal dysfunction. Meanwhile, there was no such significant difference in plasma concentrations of glutamine, glycine, and tyrosine (Tables 5 and 6), indicating that they would not be useful as markers of IR. Furthermore, phenylalanine, ornithine, and lysine did not correlate with HOMA-IR (Table 4).

CKD causes changes in different amino acids depending on the disease that caused it. For example, DN has been reported to reduce levels of serine, glycine, GABA, and tryptophan [45]. The positive correlation between 1/CysC and GABA in this study confirms this, but it is not true for other amino acids. Conversely, citrulline, cysteine, glycine, and ornithine correlated negatively with 1/CysC. Furthermore, as there was no significant difference between the control and T2DM groups in the levels of each of these amino acids (Tables 5 and 6), using them as markers of mild renal dysfunction would not be appropriate.

In recent years, DR has been reported to be a prognostic factor for progression of CKD in patients with T2DM [46], and serum creatinine is suggested as a marker of DR development in T2DM [47]. The T2DM group in this study included 19 patients with DR as comorbidity, and 78.9% of patients with DR were included in the subgroup with mild renal dysfunction. This correlation between DR and mild renal dysfunction does not contradict these findings. HT and T2DM are diseases that usually coexist [48]. However, this study was not conducted from the viewpoint of DR and/or HT; thus, further research is needed. One of the limitations of this research is the lack noninclusion of female patients in the study population. In addition, there are sex differences in BCAA metabolism arising from differences in muscle and adipose tissue [49]. Consequently, future research is needed regarding how IR and mild renal dysfunction interact with BCAA metabolism in females.

## 5. Conclusions

The results of this study showed that renal function must be considered when using plasma BCAA concentration as a marker of IR, even in mild renal dysfunction. Furthermore, the results confirm that increased alanine and glutamic acid levels indicate IR, regardless of mild renal dysfunction.

## Figures and Tables

**Table 1:** Correlation among each amino acid according to age and eGFR in control participants.

	Age		eGFR	
	Partial correlation	*p* value	Partial correlation	*p* value
Taurine	-0.6407	<0.001	0.0996	0.5006
Alanine	0.0917	0.5352	0.0201	0.8920
GABA	-0.0726	0.6238	0.0405	0.7847
Arginine	0.1128	0.4451	-0.0272	0.8546
Asparagine	0.0008	0.9956	0.0735	0.6195
Aspartic acid	-0.5445	<0.001	0.1330	0.3837
Citrulline	0.2681	0.0654	-0.1354	0.3587
Cystine	0.4780	<0.001	-0.1403	0.3415
Glutamic acid	-0.1621	0.2710	0.2234	0.1269
Glutamine	-0.0549	0.7109	-0.1239	0.4013
Glycine	-0.0844	0.5685	-0.2819	0.0523
Histidine	-0.0018	0.9905	0.0013	0.9929
Isoleucine	-0.1092	0.4600	0.0362	0.8071
Leucine	-0.2315	0.1134	0.0882	0.5512
Lysine	0.1774	0.2276	-0.0202	0.8916
Methionine	0.1759	0.2316	0.0134	0.9281
Ornithine	0.2518	0.0842	-0.1320	0.3713
Phenylalanine	0.1775	0.2274	-0.1152	0.4354
Proline	0.0604	0.6834	-0.0765	0.6051
Serine	-0.0549	0.7108	-0.0509	0.7312
Threonine	0.0648	0.6615	0.0325	0.8264
Tryptophan	0.2689	0.0646	0.2026	0.1672
Tyrosine	0.1702	0.2475	0.1185	0.4224
Valine	-0.1104	0.4550	-0.1039	0.4824
Total amino acid	-0.0058	0.9689	-0.0962	0.5152
NEAA	-0.0092	0.9505	-0.1219	0.4093
EAA	0.0039	0.9825	-0.0083	0.9555
BCAA	-0.1556	0.2910	0.0233	0.8751

eGFR: estimated glomerular filtration rate; GABA; *γ*-aminobutyric acid; NEAA: nonessential amino acid; EAA: essential amino acid; BCAA: branched-chain amino acid.

**Table 2:** Classification of medicine administered to patients with type 2 diabetes mellitus.

Biguanide	57 (61.3)
Thiazolidine derivatives	8 (8.6)
Sulfonylurea	19 (20.4)
Glinide	14 (15.1)
Dipeptidyl peptidase 4 inhibitor	73 (78.5)
*α*-Glucosidase inhibitor	27 (29.0)
Sodium glucose cotransporter 2 inhibitor	24 (25.8)

Values are presented as number (%).

**Table 3:** Distribution of HbA1c and BMI in patients with type 2 diabetes mellitus.

HbA1c (%)	Number (%)	BMI (kg/m^2^)	Number (%)
<6.0	3 (3.2)	<18.5	1 (1.1)
6.0 ≤ HbA1c < 7.0	37 (39.8)	18.5 ≤ BMI < 25.0	47 (50.5)
7.0 ≤ HbA1c < 8.0	34 (36.6)	25.0 ≤ BMI < 30.0	27 (29.0)
8.0 ≤ HbA1c < 9.0	15 (16.1)	30.0 ≤ BMI < 35.0	13 (14.0)
≥9.0	4 (4.3)	≥35.0	5 (5.4)

HbA1c: glycated hemoglobin; BMI: body mass index.

**Table 4:** Correlations between each amino acid according to age, HOMA-IR, and 1/CysC in patients with type 2 diabetes mellitus.

	Age		HOMA-IR		1/CysC	
	Partial correlation	*p* value	Partial correlation	*p* value	Partial correlation	*p* value
Taurine	-0.1188	0.2619	-0.1519	0.1507	-0.0465	0.6613
Alanine	0.0260	0.8064	0.3417	<0.001	-0.1253	0.2367
GABA	-0.1165	0.2714	0.0672	0.5268	0.4110	<0.001
Arginine	0.0265	0.8028	0.0212	0.8417	-0.0983	0.3540
Asparagine	0.0565	0.5945	-0.1687	0.1099	-0.1372	0.1947
Aspartic acid	0.0003	0.9976	0.1155	0.2756	0.0760	0.4739
Citrulline	0.0385	0.7171	-0.0379	0.7214	-0.3797	<0.001
Cystine	-0.1063	0.3157	0.0674	0.5258	-0.3688	<0.001
Glutamic acid	-0.306	0.2174	0.4978	<0.001	0.1421	0.1792
Glutamine	0.1316	0.2136	-0.2755	0.0082	-0.0775	0.4655
Glycine	-0.1210	0.2534	-0.3299	0.0014	-0.3015	0.0037
Histidine	-0.1320	0.2122	-0.0639	0.5474	-0.1437	0.1743
Isoleucine	-0.2045	0.0519	0.3793	<0.001	0.1850	0.0792
Leucine	-0.806	0.0071	0.3179	0.0021	0.1791	0.0893
Lysine	-0.0380	0.7208	-0.0426	0.6887	0.0027	0.9800
Methionine	-0.0023	0.9825	0.1945	0.0647	-0.0791	0.4562
Ornithine	0.0741	0.4853	-0.0702	0.5086	-0.3236	0.0018
Phenylalanine	0.0390	0.7139	0.1852	0.0789	-0.695	0.1083
Proline	-0.0635	0.5496	0.1375	0.1938	-0.0917	0.3872
Serine	0.0342	0.7477	0.0350	0.7416	0.0926	0.3825
Threonine	-0.0428	0.6868	0.1117	0.2916	0.0084	0.9370
Tryptophan	-0.1863	0.0771	0.1569	0.1374	0.1320	0.2124
Tyrosine	0.1249	0.2383	0.3065	0.0031	0.0547	0.6068
Valine	-0.2572	0.0139	0.3630	<0.001	0.2927	0.0049
Total amino acid	-0.0871	0.4119	0.1603	0.1291	-0.0935	0.3779
NEAA	0.0066	0.9501	0.0512	0.6297	-0.2101	0.0456
EAA	-0.2004	0.0568	0.2660	0.0108	0.1419	0.1796
BCAA	-0.2640	0.0115	0.3648	<0.001	0.2492	0.0172

HOMA-IR: homeostasis model assessment of insulin resistance; CysC: cystatin C; GABA: *γ*-aminobutyric acid; NEAA; nonessential amino acid; EAA; essential amino acid; BCAA: branched-chain amino acid.

**Table 5:** Plasma amino acid concentrations in each group.

	C (nmol/mL) (*n* = 49)	IR-L (nmol/mL) (*n* = 31)	IR-M (nmol/mL) (*n* = 26)	IR-H (nmol/mL) (*n* = 37)
Alanine	335.3 ± 91.7	347.1 ± 92.7	409.5 ± 106.5	440.6 ± 85.8
Glutamic acid	46.4 ± 14.2	56.2 ± 30.7	61.7 ± 14.4	76.3 ± 23.0
Glutamine	575.0 ± 67.5	585.0 ± 120.0	600.2 ± 86.3	551.8 ± 81.5
Glycine	204.3 ± 37.4	213.3 ± 42.7	207 ± 43.8	189.0 ± 35.2
Tyrosine	65.1 ± 11.0	65.1 ± 27.9	66.2 ± 14.8	69.7 ± 14.8

Values are presented as mean ± standard deviation. C: control group; IR-L, T2DM group with HOMA − IR ≤ 1.60; HM-M, T2DM group with 1.60 < HOMA − IR < 2.05; IR-H, T2DM group with HOMA − IR ≥ 2.50; T2DM: type 2 diabetes mellitus; HOMA-IR; homeostasis model assessment of insulin resistance.

**Table 6:** Probability of comparison among groups for each amino acid (Table 5).

	C vs. IR-L	C vs. IR-M	C vs. IR-H
Alanine	0.946	0.037	<0.001
Glutamic acid	0.801	0.006	<0.001
Glutamine	0.840	0.801	0.741
Glycine	0.823	1.000	0.431
Tyrosine	0.549	1.000	0.608

C: control group; IR-L, T2DM group with HOMA − IR ≤ 1.60; HM-M, T2DM group with 1.60 < HOMA − IR < 2.05; IR-H, T2DM group with HOMA − IR ≥ 2.50; T2DM: type 2 diabetes mellitus; HOMA-IR: homeostasis model assessment of insulin resistance.

**Table 7:** Plasma amino acid concentrations in each group.

	C (nmol/mL) (*n* = 25)	CysC-L (nmol/mL) (*n* = 48)	CysC-H (nmol/mL) (*n* = 46)
GABA	19.5 ± 5.62	23.5 ± 9.26	19.5 ± 6.70
Citrulline	31.3 ± 7.72	27.5 ± 10.1	33.5 ± 13.0
Cystine	34.0 ± 9.35	31.8 ± 7.12	37.3 ± 11.3
Glycine	207.6 ± 46.4	196.5 ± 37.7	207.3 ± 44.1
Ornithine	58.2 ± 10.4	56.2 ± 11.1	62.2 ± 15.7

Values are presented as mean ± standard deviation. C: control group; CysC-L, T2DM group with CysC < 0.95; CysC-H, T2DM group with CysC ≥ 0.95; CysC: cystatin C; GABA: *γ*-aminobutyric acid; T2DM: type 2 diabetes mellitus.

**Table 8:** Probability of comparison among groups for each amino acid (Table 7).

	C vs. CysC-L	C vs. CysC-H	CysC-L vs. CysC-H
GABA	0.0372	0.9910	0.0918
Citrulline	0.0626	0.8703	0.0652
Cystine	0.4784	0.3987	0.0249
Glycine	0.6230	0.9613	0.5052
Ornithine	0.4515	0.7483	0.2666

C: control group; CysC-L, T2DM group with CysC < 0.93; CysC-H, T2DM group with CysC ≥ 0.93; CysC: cystatin C; GABA: *γ*-aminobutyric acid; T2DM: type 2 diabetes mellitus.

## Data Availability

The data sets used or analyzed in this study are available from the author on reasonable request.

## References

[1] Wang T. J., Larson M. G., Vasan R. S. (2011). Metabolite profiles and the risk of developing diabetes. *Nature Medicine*.

[2] Yamakado M., Nagao K., Imaizumi A. (2015). Plasma Free Amino Acid Profiles Predict Four-Year Risk of Developing Diabetes, Metabolic Syndrome, Dyslipidemia and Hypertension in Japanese Population. *Scientific Reports*.

[3] Bloomgarden Z. (2018). Diabetes and branched-chain amino acids: what is the link?. *Journal of Diabetes*.

[4] Saleem T., Dahpy M., Ezzat G., Abdelrahman G., Abdel-Aziz E., Farghaly R. (2019). The profile of plasma free amino acids in type 2 diabetes mellitus with insulin resistance: association with microalbuminuria and macroalbuminuria. *Applied Biochemistry and Biotechnology*.

[5] Liu X., Zheng Y., Guasch-Ferré M. (2019). High plasma glutamate and low glutamine-to-glutamate ratio are associated with type 2 diabetes: case-cohort study within the PREDIMED trial. *Nutrition, Metabolism, and Cardiovascular Diseases*.

[6] Matsuoka K., Kato K., Takao T. (2017). Concentrations of various tryptophan metabolites are higher in patients with diabetes mellitus than in healthy aged male adults. *Diabetology International*.

[7] Cheng S., Rhee E. P., Larson M. G. (2012). Metabolite profiling identifies pathways associated with metabolic risk in humans. *Circulation*.

[8] Jenstad M., Chaudhry F. A. (2013). The amino acid transporters of the glutamate/GABA-glutamine cycle and their impact on insulin and glucagon secretion. *Frontiers in Endocrinology*.

[9] Lynch C. J., Adams S. H. (2014). Branched-chain amino acids in metabolic signalling and insulin resistance. *Nature Reviews Endocrinology*.

[10] Kawanaka M., Nishino K., Oka T. (2015). Tyrosine levels are associated with insulin resistance in patients with nonalcoholic fatty liver disease. *Hepatic Medicine: Evidence and Research*.

[11] Seibert R., Abbasi F., Hantash F. M., Caulfield M. P., Reaven G., Kim S. H. (2015). Relationship between insulin resistance and amino acids in women and men. *Physiological Reports*.

[12] Adeva M., Calviño J., Souto G., Donapetry C. (2012). Insulin resistance and the metabolism of branched-chain amino acids in humans. *Amino Acids*.

[13] McCormack S. E., Shaham O., McCarthy A. (2013). Circulating branched-chain amino acid concentrations are associated with obesity and future insulin resistance in children and adolescents. *Pediatric Obesity*.

[14] Vanweert F., de Ligt M., Hoeks J., Hesselink M. K. C., Schrauwen P., Phielix E. (2021). Elevated plasma branched-chain amino acid levels correlate with type 2 diabetes-related metabolic disturbances. *Journal of Clinical Endocrinology and Metabolism*.

[15] Connelly M. A., Wolak-Dinsmore J., Dullaart R. P. F. (2017). Branched chain amino acids are associated with insulin resistance independent of leptin and adiponectin in subjects with varying degrees of glucose tolerance. *Metabolic Syndrome and Related Disorders*.

[16] Toth-Manikowski S., Atta M. G. (2015). Diabetic kidney disease: pathophysiology and therapeutic targets. *Journal of Diabetes Research*.

[17] The Japanese Society for Dialysis Therapy. https://docs.jsdt.or.jp/overview/file/2018/pdf/03.pdf.

[18] van de Poll M. C. G., Soeters P. B., Deutz N. E. P., Fearon K. C. H., Dejong C. H. C. (2004). Renal metabolism of amino acids: its role in interorgan amino acid exchange. *American Journal of Clinical Nutrition*.

[19] Verrey F., Ristic Z., Romeo E. (2005). Novel renal amino acid transporters. *Annual Review of Physiology*.

[20] Holeček M. (2018). Branched-chain amino acids in health and disease: metabolism, alterations in blood plasma, and as supplements. *Nutrition and Metabolism*.

[21] Garibotto G., Sofia A., Saffioti S., Bonanni A., Mannucci I., Verzola D. (2010). Amino acid and protein metabolism in the human kidney and in patients with chronic kidney disease. *Clinical Nutrition*.

[22] Tizianello A., Deferrari G., Garibotto G. (1983). Branched-chain amino acid metabolism in chronic renal failure. *Kidney International. Supplement*.

[23] Bergström J., Alvestrand A., Fürst P. (1990). Plasma and muscle free amino acids in maintenance hemodialysis patients without protein malnutrition. *Kidney International*.

[24] Li R., Dai J., Kang H. (2018). The construction of a panel of serum amino acids for the identification of early chronic kidney disease patients. *Journal of Clinical Laboratory Analysis*.

[25] Mahbub M. H., Yamaguchi N., Nakagami Y. (2021). Association of plasma branched-chain and aromatic amino acids with reduction in kidney function evaluated in apparently healthy adults. *Journal of Clinical Medicine*.

[26] Kopple J. D. (2007). Phenylalanine and tyrosine metabolism in chronic kidney failure. *Journal of Nutrition*.

[27] Harper A. E., Miller R. H., Block K. P. (1984). Branched-chain amino acid metabolism. *Annual Review of Nutrition*.

[28] Matthews D. R., Hosker J. P., Rudenski A. S., Naylor B. A., Treacher D. F., Turner R. C. (1985). Homeostasis model assessment: insulin resistance and beta-cell function from fasting plasma glucose and insulin concentrations in man. *Diabetologia*.

[29] Japan Diabetes Society (2010). *Treatment guide for diabetes 2010*.

[30] Yamada C., Mitsuhashi T., Hiratsuka N., Inabe F., Araida N., Takahashi E. (2011). Optimal reference interval for homeostasis model assessment of insulin resistance in a Japanese population. *Journal of Diabetes Investigation*.

[31] Abrahamson M., Olafsson I., Palsdottir A. (1990). Structure and expression of the human cystatin C gene. *Biochemical Journal*.

[32] Hoek F. J., Kemperman F. A. W., Krediet R. T. (2003). A comparison between cystatin C, plasma creatinine and the Cockcroft and Gault formula for the estimation of glomerular filtration rate. *Nephrology, Dialysis, Transplantation*.

[33] Laterza O. F., Price C. P., Scott M. G. (2002). Cystatin C: an improved estimator of glomerular filtration rate?. *Clinical Chemistry*.

[34] Pucci L., Triscornia D., Lucchesi S. (2007). Cystatin C and estimates of renal function: searching for a better measure of kidney function in diabetic patients. *Clinical Chemistry*.

[35] Matsuo S., Imai E., Horio M., Yokoyama H., Hishida A. (2009). Revised equations for estimated GFR from serum creatinine in Japan. *American Journal of Kidney Diseases*.

[36] Ministry of Health, Labour and Welfare of Japan (2015). Ethical guidelines for medical and health research involving human subjects. https://www.mhlw.go.jp/file/06-Seisakujouhou-10600000-Daijinkanboukouseikagakuka/0000080278.pdf.

[37] The International Society of Nephrology (2013). KDIGO 2012 clinical practice guideline for the evaluation and management of chronic kidney disease. *Kidney International*.

[38] Japan Society for the Study of Obesity (2016). *Guidelines for the management of obesity disease 2016*.

[39] Zitta S., Schrabmair W., Reibnegger G. (2013). Glomerular filtration rate (GFR) determination via individual kinetics of the inulin-like polyfructosan sinistrin versus creatinine-based population-derived regression formulae. *BMC Nephrology*.

[40] Cheuiche A. V., Queiroz M., Azeredo-da-Silva A. L. F., Silveiro S. P. (2019). Performance of cystatin C-based equations for estimation of glomerular filtration rate in diabetes patients: a PRISMA-compliant systematic review and meta-analysis. *Scientific Reports*.

[41] Chen S., Akter S., Kuwahara K. (2019). Serum amino acid profiles and risk of type 2 diabetes among Japanese adults in the Hitachi Health Study. *Scientific Reports*.

[42] Gao X., Hou R., Li X. (2021). The association between leucine and diabetic nephropathy in different gender: a cross-sectional study in Chinese patients with type 2 diabetes. *Frontiers in Endocrinology*.

[43] Nie C., He T., Zhang W., Zhang G., Ma X. (2018). Branched chain amino acids: beyond nutrition metabolism. *International Journal of Molecular Sciences*.

[44] Luo H., Li J., Feng X. F. (2020). Plasma phenylalanine and tyrosine and their interactions with diabetic nephropathy for risk of diabetic retinopathy in type 2 diabetes. *BMJ Open Diabetes Research & Care*.

[45] Zeng L., Yu Y., Cai X. (2019). Differences in serum amino acid phenotypes among patients with diabetic nephropathy, hypertensive nephropathy, and chronic nephritis. *Medical Science Monitor*.

[46] Park H. C., Lee Y. K., Cho A. (2019). Diabetic retinopathy is a prognostic factor for progression of chronic kidney disease in the patients with type 2 diabetes mellitus. *PLoS One*.

[47] Yun J. H., Kim J. M., Jeon H. J., Oh T., Choi H. J., Kim B. J. (2020). Metabolomics profiles associated with diabetic retinopathy in type 2 diabetes patients. *PLoS One*.

[48] Epstein M., Sowers J. R. (1992). Diabetes mellitus and hypertension. *Hypertension*.

[49] Honda T., Kobayashi Y., Togashi K. (2016). Associations among circulating branched-chain amino acids and tyrosine with muscle volume and glucose metabolism in individuals without diabetes. *Nutrition*.

